# Treatment of colitis cystica profunda via endoscopic submucosal dissection

**DOI:** 10.1055/a-2744-8895

**Published:** 2025-12-12

**Authors:** Xueying Lai, Yiwei He, Bin Liu, Hui Deng

**Affiliations:** 1 477093 Department of Gastroenterology, The Affiliated Panyu Central Hospital, Guangzhou Medical University, Guangzhou, China; 2 198153 Department of Pharmacy, Nanfang Hospital, Southern Medical University, Guangzhou, China

A 47-year-old asymptomatic male patient underwent a colonoscopy during a physical examination, which revealed a 15 mm × 25 mm raised lesion in the rectum ( Fig. 1 ). Endoscopic ultrasonography showed a 14 mm anechoic focus within the lesion, with posterior acoustic enhancement ( Fig. 2 **a** ). Contrast-enhanced computed tomography revealed a hypodense lesion in the rectum with well-defined borders but heterogeneous density, demonstrating mild heterogeneous enhancement ( Fig. 2 **b** ). After obtaining informed consent from the patient, endoscopic submucosal dissection (ESD) was performed on the lesion ( Video 1 ), and a 45 mm × 52 mm specimen was resected ( Fig. 3 , Fig. 4 ). Histological examination revealed colitis cystica profunda (CCP) with focal rupture of cystic lesions ( Fig. 5 ). The patient recovered well after ESD and was discharged on the third day without complaints.

**Fig. 1:**
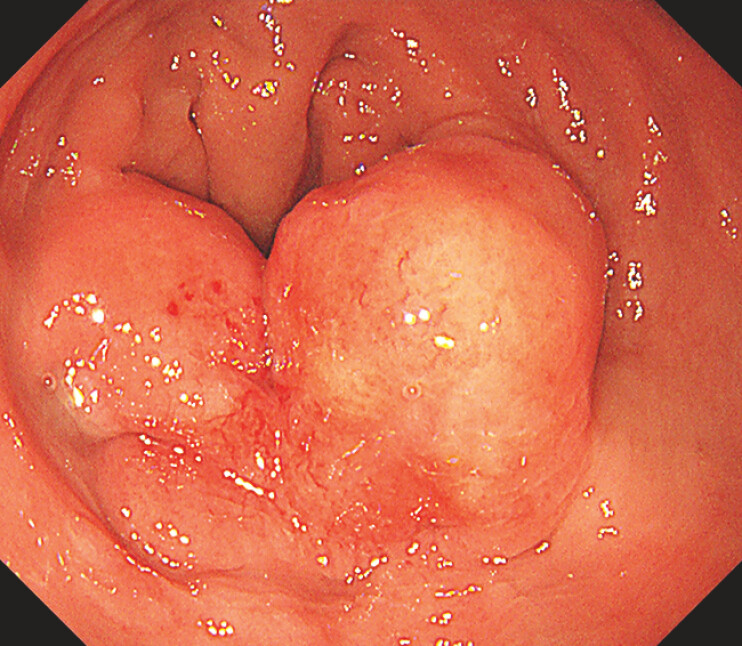
Endoscopy showed a 15 mm × 25 mm raised lesion in the rectum. The lesion had poorly defined borders, with a smooth, erythematous mucosal surface. One side of the lesion showed significant mucosal elevation, while the other side was slightly depressed.

**Fig. 2:**
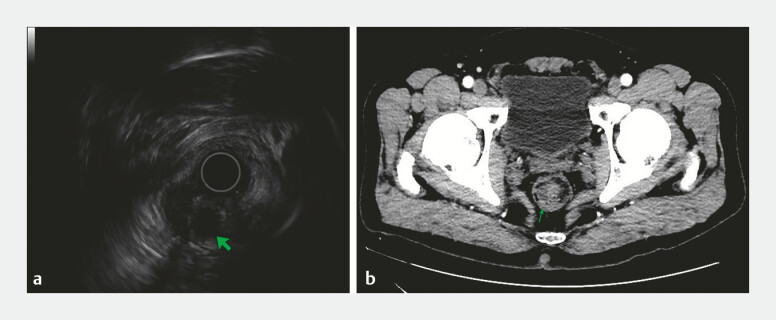
**a** Endoscopic ultrasonography showed a 14 mm anechoic focus within the lesion, with posterior acoustic enhancement. **b** Contrast-enhanced computed tomography revealed a hypodense lesion in the rectum with well-defined borders but heterogeneous density, demonstrating mild heterogeneous enhancement.

**Fig. 3:**
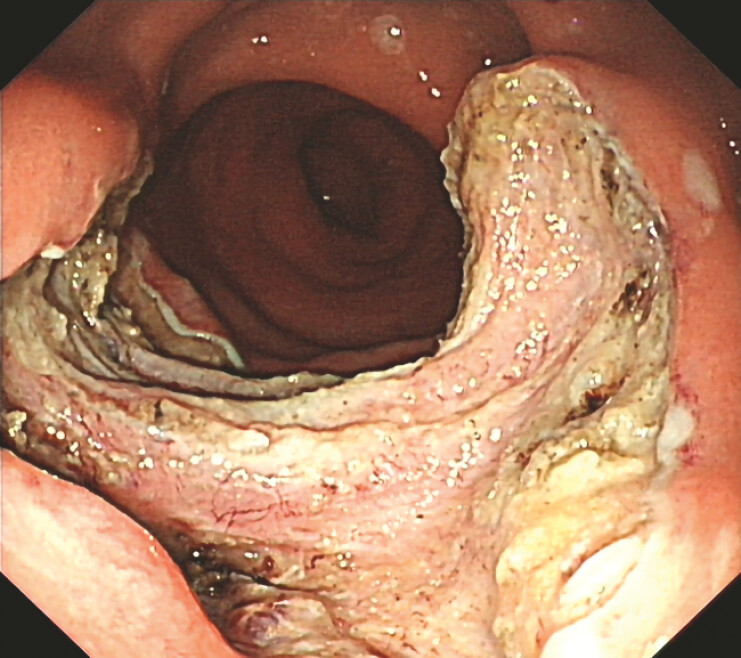
The mucosal wound after endoscopic submucosal dissection.

**Fig. 4:**
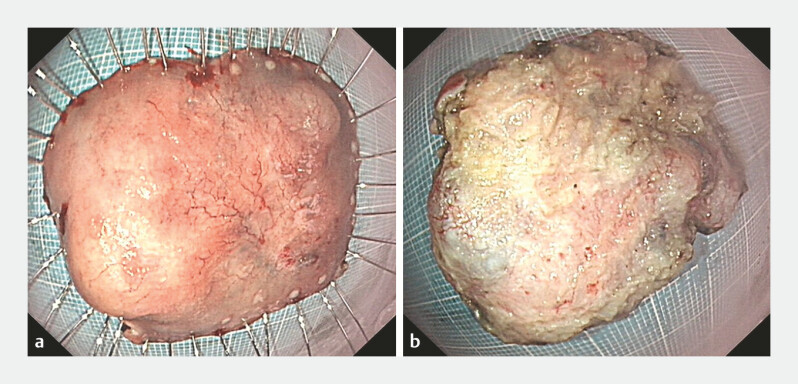
The resected specimen, approximately 45 mm × 52 mm in size. The specimen contained two firm, raised nodules, 20 and 8 mm in diameters. **a** An anterior view of the specimen. **b** A posterior view of the specimen.

**Fig. 5:**
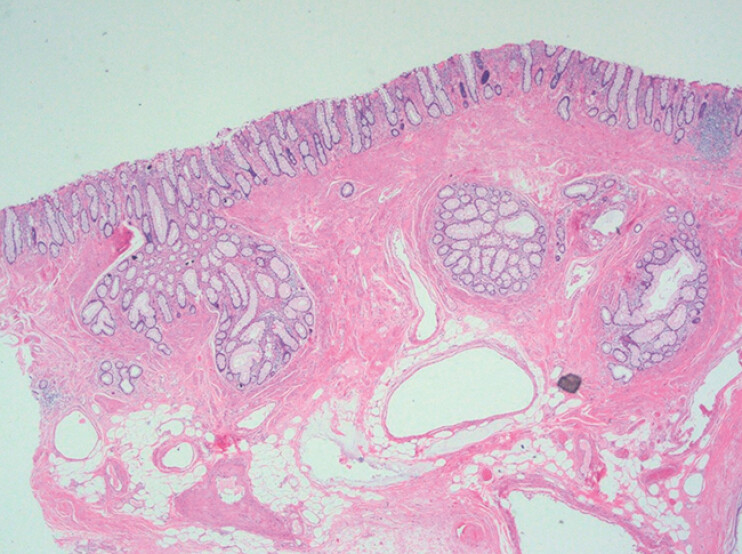
Hematoxylin and eosin staining of the resected specimen showed ectopic epithelium within the lamina propria or submucosa, forming retention mucus cysts. Focal cyst rupture with associated mucus extravasation was observed.

Treatment of colitis cystica profunda via endoscopic submucosal dissection.Video 1

CCP is a non-neoplastic disorder characterized by the presence of mucus-containing cysts within the submucosa of the colon and rectum 1 . It is believed that the etiology of CCP is chronic prolapse, leading to inflammation and mucosal ulceration, followed by epithelial implantation and the formation of rectal submucosal cysts 2 . Owing to its low incidence and the lack of specific clinical and endoscopic manifestations, it is often misdiagnosed as other neoplastic space-occupying lesions in the intestine. Consequently, previous studies have primarily reported surgical resection as the mainstay of treatment for CCP. In the present case, the patient was treated with ESD, a minimally invasive approach compared to surgical resection. Our experience demonstrates that ESD can be employed as a viable endoscopic technique for diagnosing and treating CCP, thereby reducing unnecessary surgical interventions.

Endoscopy_UCTN_Code_TTT_1AQ_2AD_3AD

## Notes

Endoscopy E-Videos https://eref.thieme.de/e-videos
*E-Videos* is an open access online section of the journal *Endoscopy* , reporting on interesting cases and new techniques in gastroenterological endoscopy. All papers include a high-quality video and are published with a Creative Commons CC-BY license. Endoscopy E-Videos qualify for HINARI discounts and waivers and eligibility is automatically checked during the submission process. We grant 100% waivers to articles whose corresponding authors are based in Group A countries and 50% waivers to those who are based in Group B countries as classified by Research4Life (see: https://www.research4life.org/access/eligibility/ ). This section has its own submission website at https://mc.manuscriptcentral.com/e-videos .
